# Help the Defectives

**Published:** 1906-06-02

**Authors:** 


### HELP THE DEFECTIVES.

Dr. Shuttleworth, formerly medical examiner of defective children, under the School Board for London, with Mrs. Chamberlain, Sir Noel Walker, Sir Arthur Clay, and the Vicar of St. Jude's, South Kensington, are making strenuous efforts to organise and develop a scheme to help the disabled in co-operation with several existing societies for the mentally and physically defective. The method pursued is to enlist the aid of relatives, private charity, and the State, to secure the training and employment of the defective and the after-care of those leaving special schools where such children are now provided for by the education authorities. Another branch of the work which we would highly commend is the attempt to brighten by occupation the lives of those who can only work at home, and to help them to maintain themselves in comfort. Already some 600 cases have been visited, and the need of the moment is a central employment room and meeting place for helpers and those who need assistance. The total annual expenditure is estimated at only £150 to provide the required help and supply the necessary supervision. We have no doubt several of our readers will be glad to hear of the movement, and to offer their co-operation either in money or personal service by communicating with Miss Arnould, Hon. Secretary, 9 Nevern Square, S.W.

